# A Critical Evaluation of the Biological Construct Skeletal Muscle Hypertrophy: Size Matters but So Does the Measurement

**DOI:** 10.3389/fphys.2019.00247

**Published:** 2019-03-12

**Authors:** Cody T. Haun, Christopher G. Vann, Brandon M. Roberts, Andrew D. Vigotsky, Brad J. Schoenfeld, Michael D. Roberts

**Affiliations:** ^1^Department of Exercise Science, LaGrange College, LaGrange, GA, United States; ^2^School of Kinesiology, Auburn University, Auburn, AL, United States; ^3^Department of Cell, Developmental and Integrative Biology, University of Alabama at Birmingham, Birmingham, AL, United States; ^4^Department of Biomedical Engineering, Northwestern University, Evanston, IL, United States; ^5^Department of Health Sciences, CUNY Lehman College, Bronx, NY, United States

**Keywords:** myofibrillar protein, sarcoplasmic protein, fiber cross-sectional area, ultrasound, dual x-ray absorptiometry, muscle hypertrophy, resistance exercise, skeletal muscle

## Abstract

Skeletal muscle is highly adaptable and has consistently been shown to morphologically respond to exercise training. Skeletal muscle growth during periods of resistance training has traditionally been referred to as skeletal muscle hypertrophy, and this manifests as increases in muscle mass, muscle thickness, muscle area, muscle volume, and muscle fiber cross-sectional area (fCSA). Delicate electron microscopy and biochemical techniques have also been used to demonstrate that resistance exercise promotes ultrastructural adaptations within muscle fibers. Decades of research in this area of exercise physiology have promulgated a widespread hypothetical model of training-induced skeletal muscle hypertrophy; specifically, fCSA increases are accompanied by proportional increases in myofibrillar protein, leading to an expansion in the number of sarcomeres in parallel and/or an increase in myofibril number. However, there is ample evidence to suggest that myofibrillar protein concentration may be diluted through sarcoplasmic expansion as fCSA increases occur. Furthermore, and perhaps more problematic, are numerous investigations reporting that pre-to-post training change scores in macroscopic, microscopic, and molecular variables supporting this model are often poorly associated with one another. The current review first provides a brief description of skeletal muscle composition and structure. We then provide a historical overview of muscle hypertrophy assessment. Next, current-day methods commonly used to assess skeletal muscle hypertrophy at the biochemical, ultramicroscopic, microscopic, macroscopic, and whole-body levels in response to training are examined. Data from our laboratory, and others, demonstrating correlations (or the lack thereof) between these variables are also presented, and reasons for comparative discrepancies are discussed with particular attention directed to studies reporting ultrastructural and muscle protein concentration alterations. Finally, we critically evaluate the biological construct of skeletal muscle hypertrophy, propose potential operational definitions, and provide suggestions for consideration in hopes of guiding future research in this area.

## Introduction

Etymology of the term *hypertrophy* reveals derivation from the English term “*hyper-*,” denoting “above” or “beyond,” and Greek term “*-trophia*,” denoting “growth” or “nourishment.” In the context of resistance training, skeletal muscle hypertrophy has been generally defined as an increase in muscle mass and cross-sectional area (CSA) at the whole tissue and cellular levels (Russell et al., 2000). Historically, muscle hypertrophy has been posited to occur in response to the accrual of contractile or structural proteins due to an increase in the number of sarcomeres in parallel in pre-existing myofibrils of muscle fibers, which results in an increase in fiber cross-sectional area (fCSA) (Russell et al., 2000). Accordingly, it is logical to assume that investigations reporting increases in muscle size and myofibrillar protein alterations would clearly demonstrate this phenomenon. However, while skeletal muscle hypertrophy is considered a hallmark adaptation of resistance training of sufficient duration, there have been inconsistent observations in the scientific literature dependent upon the outcome variables being reported. This, in part, is likely due to numerous methods being used to assess skeletal muscle hypertrophy, and each of these methods assess distinct characteristics of skeletal muscle. Many studies in the literature detect regional adaptations using dual-energy x-ray absorptiometry (DXA), computed tomography (CT) scanning, magnetic resonance imaging (MRI), and/or ultrasound assessment. Additionally, the percutaneous muscle biopsy technique has allowed exercise scientists to examine pre- and post-intervention differences in muscle fCSA. While these techniques have provided excellent insight regarding how exercise training affects body composition and muscle tissue morphology, recent work from our laboratories, and that of others, suggest that pre- to post-training change scores in these measurements often correlate poorly with one another.

We are not the first to propose that measures and scales of muscle growth are not strongly related. For instance, Dr. Edward E. Gordon published a commentary by Gordon (1967) stating:

*First, what is meant by hypertrophy: increase in girth of a limb, volume of a muscle, or the related weight? Or, are we referring to the individual muscle fiber, the smallest anatomical unit of muscle? In* [hypertrophy], *the gross and microscopic dimensions are regarded as running parallel courses, and therefore, as being interchangeable. There would be no serious error if in hypertrophy the sum of the enlarged parts equalled an increased whole. But such an equation is not always found.* (p. 129)

Gordon went on to note these discrepancies in his own research and in the work from some of his contemporaries, stating that there appears to be “*a complete dissociation between whole muscle and fiber size in trained animals*” (p. 130). Indeed, historical literature is rife with examples that further support this narrative. For example, in the first formal study on work-induced hypertrophy, Morpurgo reported 26% greater fCSA values in run-trained versus untrained animals, although only a 13% increase in whole-muscle CSA was observed in the former group (Morpurgo, 1897). Morpurgo related this finding to an increase in sarcoplasmic volume [i.e., muscle intracellular fluid (ICF)], rather than an increase in muscle fiber or myofibril number. Likewise, decades later, Goldspink reported that 5 weeks of resistance-like training of the biceps muscles in mice produced a 30% increase in fCSA relative to untrained, age-matched controls, whereas muscle weights in both groups were nearly identical (Goldspink, 1964). Other historical reports of biochemical and ultrastructural changes underpinning changes in muscle size have been inconsistent, which are discussed in following sections.

The aforementioned evidence calls into question what is meant by the term skeletal muscle hypertrophy. We feel a critical evaluation of the term and the construct validity of assessments employed warrant special consideration by researchers moving forward in this area of inquiry. In particular, researchers invoking the term should agree on an operational definition so that the construct validity of an assessment or assessments can be better characterized and adopted for future research endeavors. Therefore, we begin this review by briefly describing skeletal muscle composition and structure, and provide a historical overview of the scientific assessment of muscle hypertrophy. Next, we discuss laboratory-based measurements used to assess training-induced skeletal muscle hypertrophy at the biochemical, ultrastructural, histological, and gross anatomical levels, and highlight the strengths and limitations of these approaches as well as how they differ from one another. We then present whole-body, whole-tissue, microscopic, and biochemical assessments of skeletal muscle hypertrophy obtained by our laboratory, and others’, which demonstrate the degree of agreeability – or lack thereof – between methods with particular attention directed to muscle protein concentration alterations. Finally, we provide potential operational definitions, suggestions for future research, and discuss methods that could be adopted to more accurately assess the biological construct of skeletal muscle hypertrophy. While this review is centered on empirical data obtained in humans, reference to pertinent animal models are also provided where applicable given that this area of human physiology has been largely preceded by delicate animal work.

## Skeletal Muscle Composition and Structure

**FIGURE 1:**
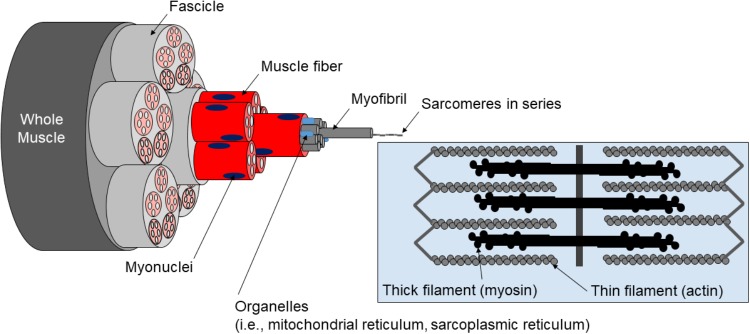
Hierarchical structure of muscle. Pictured is the hierarchical structure of muscle described in text.

Skeletal muscle tissue can be categorized into the following levels of organization: (a) whole muscle sheathed by fascia (i.e., epimysium), (b) muscle fibers within fascicle bundles (i.e., peri- and endomysium), (c) myofibrils within individual muscle fibers, (d) sarcomeres within individual myofibrils, and (e) proteins (e.g., actin, myosin, and titin) within individual sarcomeres (Figure 1).

**FIGURE 2:**
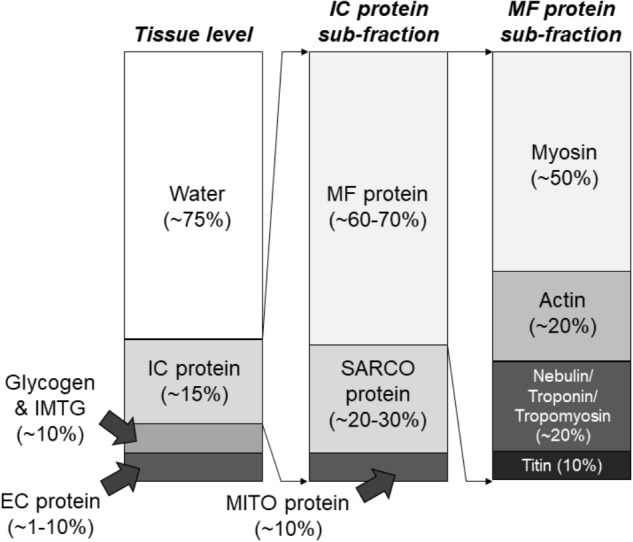
Composition of skeletal muscle tissue. These composition estimates are based upon numerous studies which have utilized biochemical and proteomics-based assessments described in text. IMTG, intramuscular triglycerides; EC, extracellular; IC, intracellular; MF, myofibrillar; SARCO, sarcoplasmic; MITO, mitochondrial.

Whole skeletal muscle is sheathed with connective tissue, primarily composed of collagen protein, and is ∼75% fluid by volume (Kjaer, 2004). Skeletal muscle can be separated into intracellular (i.e., beneath the muscle fiber membrane) and extracellular (i.e., outside the muscle fiber membrane) components. The extracellular component is primarily composed of connective tissue and vasculature, and connective tissue generally occupies between ∼1–20% of human skeletal muscle which further separates muscle into fascicular bundles of fibers and individual muscle fibers (Kjaer, 2004). Notably, the connective tissue component is also adaptable and can vary in its contribution to skeletal muscle size and strength. Muscle fibers consist primarily of myofibrils, mitochondria, and a specialized structure known as the sarcoplasmic reticulum. These are the three major components of muscle fibers (Lindstedt et al., 1998), although glycogen also constitutes ∼2–3% and intramuscular triglycerides ∼5% on average (van Loon et al., 2003; Gallagher et al., 2005). The human skeletal muscle proteome was recently “reappraised” by Gonzalez-Freire et al. (2017), who employed sensitive mass spectrometry-based techniques. Based on this analysis, most of the proteins in skeletal muscle, by percentage, are involved in metabolic processes rather contraction directly. This counters the assumption that most of the proteins in skeletal muscle serve a direct contractile role. The authors also categorized around 40% of the total number of proteins in skeletal muscle as enzymes and under 10% as contractile. Furthermore, ∼20% of proteins were characterized as mitochondrial apparently serving roles in oxidative metabolism. Notably, these percentages are relative to the total number of proteins in human skeletal muscle, and not the *concentration* of proteins within skeletal muscle. Traditionally, ∼60–70% of the human skeletal muscle mixed protein pool has been characterized as myofibrillar, ∼20–30% as sarcoplasmic, and ∼5–10% as mitochondrial (Haus et al., 2007). Other estimates suggest that myosin represents ∼50% of myofibrillar protein concentration and actin ∼20% (Yates and Greaser, 1983; Ingalls et al., 1998). Based on data from Wang (1982) and Yates and Greaser (1983), titin typically represents ∼10% of myofibrillar protein while nebulin, troponin, and tropomyosin each represent ∼5%. Quantitatively, these proteins seem to represent ∼95% of all myofibrillar proteins by concentration. Mitochondrial, sarcoplasmic reticulum, and t-tubule proteins have been estimated to occupy most of the remaining mixed protein pool, while glycolytic enzymes and other protein constituents of the sarcoplasm predominate the remaining pool (Hoppeler and Lindstedt, 1985; Al-Qusairi and Laporte, 2011). Figure 2 summarizes the percentage breakdown of these components within muscle fibers.

Considering the composition and organization of skeletal muscle tissue, it seems logical that training-induced increases in fCSA would result in proportional increases in myofibrillar protein abundance where concentrations would be largely preserved. Indeed, since ∼60–70% of muscle protein is made up of myofibrillar proteins, a number of authors have posited that skeletal muscle hypertrophy in response to resistance training is due to an increase in myofibrillar protein abundance and an increase in the number of sarcomeres in parallel in existent myofibrils (e.g., sarcomerogenesis) or newly synthesized myofibrils of existent muscle fibers (e.g., myofibrillogenesis) (Paul and Rosenthal, 2002; Schoenfeld, 2010; Wisdom et al., 2015; Franchi et al., 2017). For example, Damas et al. (2018) recently defined “true” hypertrophy as an “… accumulation of contractile and structural muscle proteins adding sarcomeres in parallel to muscle fibers” (p. 487). However, this mode of skeletal muscle hypertrophy in response to resistance training has strikingly little direct supportive evidence in human skeletal muscle samples. To the contrary, select evidence suggests a dilution of myofibrillar protein in response to short-term resistance training which is described in later sections. To better understand how we have arrived at the current hypothesized model of training-induced hypertrophy, the following section provides a brief historical overview of the assessment of skeletal muscle hypertrophy. Thenceforth, a survey of current-day methods and more detailed discussion of the biological construct of muscle hypertrophy and future directions follows.

## Historical Assessment of Skeletal Muscle Hypertrophy

Morpurgo (1897) was the first researcher to experimentally observe work-induced skeletal muscle hypertrophy in dog sartorius muscle following 2 months of run training, which he described as an increase in fiber diameter due to an increase in sarcoplasmic volume. Following Morpurgo’s seminal work, alternative definitions and modes of work-induced skeletal muscle hypertrophy emerged. For example, Helander (1961) reported greater increases in muscle weights and myofibrillar density in guinea pigs following regimented run training performed 6 days per week for ∼4 months compared to control or restricted-activity animals. Helander concluded, “Exercise thus enhances the myofilamental density in the muscle cell…” (p. 482), whereas restricted activity “… reduces its myofilamental density and instead increases sarcoplasmic content” (p. 482). Goldspink (1964) published a report that involved training mice through a pulley apparatus designed to tax the biceps brachii muscle. Following 25 days of training, mice were euthanized and muscle was prepared for the histological assessment of muscle fiber size and myofibril number per fiber in cross section. The author noted a very strong correlation between myofibril number per fiber and muscle fiber size, and this finding – along with Helander’s – helped shape the current-day consensus that increases in fCSA and myofibrillar protein accretion are proportional during resistance training. Other animal trials in the 20th century agreed with or refuted these findings (Gordon et al., 1967; Seiden, 1976), and Goldspink and Howells (1974) associated disparate findings on specific modes of hypertrophy with large variations in experimental design, and specifically, differences in the dose of exercise.

The introduction of the Duchenne biopsy needle allowed for percutaneous open biopsies to be obtained from humans (Parent, 2005). While isolated papers in the 1950s reported the allocation of a biopsy technique in humans, it was not until Jonas Bergstrom introduced his needle sampling technique in 1962 that these methods were utilized more openly in human research related to skeletal muscle hypertrophy (Ekblom, 2017). Penman (1969) was the first to characterize ultrastructural alterations in human skeletal muscle tissue in response to three forms of exercise training which were categorized as isotonic, isometric, or run training. Interestingly, all three modes of training reduced the concentration of “myosin fibers,” which Penman characterized as the number of myofibrils within a 5 μm^2^ area of muscle fibers. In a subsequent 1970 exercise training investigation, Penman reported ∼40% increases in maximum strength, although he also observed reductions in the distance between myosin filaments and modest reductions in gross cell diameter following 10 weeks of training which involved 5 sessions per week of both leg extensions and running exercise (Penman, 1970). Penman interpreted these findings to indicate that increased strength of skeletal muscle in response to resistance training without an increase in fiber size involved an increased “packing density” of contractile elements. Although pioneering, Penman’s 1969 investigation included only 6 subjects (2 in each condition), and 3 subjects in the 1970 investigation, prohibiting sufficient statistical power for population-wide inference. Following Penman’s work, a seminal 1982 paper by MacDougall et al. (1982) used transmission electron microscopy (TEM) methods and reported *reductions* in biceps brachii myofibrillar and mitochondrial volumes as well as an increase in sarcoplasmic volume in response to 6 months of resistance training in previously untrained human subjects. Furthermore, muscle samples from a group of seven bodybuilders and powerlifters were compared to the previously untrained subjects who underwent 6 months of resistance training, and authors reported that this analysis revealed *lower* myofibrillar volume and *greater* sarcoplasmic volume in the fibers of bodybuilder and powerlifter subjects. These authors provocatively concluded that decrements in myofibrillar volume with increased resistance training experience may have been related to greater glycogen accumulation and/or increases in ICF beyond that related to glycogen accumulation (e.g., ∼3 g of water/1 g of stored glycogen, increased ion or organelle abundance), particularly in the bodybuilder and powerlifter subjects. Notwithstanding, the contribution of sarcoplasmic constituents to muscle fiber hypertrophy during resistance training has remained largely unexplored.

Between 1970 and 1990, studies by Gollnick, Saltin, Tesch, Staron, Sale, MacDougall, Alway, and others utilized the Bergstrom technique to histologically evaluate muscle fiber type composition and fCSA differences between well-trained weightlifters and untrained subjects (Gollnick et al., 1972; Staron et al., 1984; Tesch et al., 1984; Larsson and Tesch, 1986; Sale et al., 1987; Alway et al., 1988). While most of these studies examined the vastus lateralis (VL), some studies biopsied the deltoid, soleus, biceps brachii, or trapezius muscles. In the 1990s, several exercise physiology laboratories sought to determine how weeks to months of resistance training affected VL muscle fCSA values in untrained individuals. Notably, Staron, Hikida, and many others performed seminal work in this area (Staron et al., 1990, 1991, 1994; Wang et al., 1993), and this research has been carried on by several other laboratories in the 21st century (Hikida et al., 2000; Kadi et al., 2004; Kim et al., 2005; Mackey et al., 2007, 2011; Petrella et al., 2008; Mitchell et al., 2012; Snijders et al., 2016; Stec et al., 2016; Reidy et al., 2017b; Mobley et al., 2018). Collectively, most of these studies have demonstrated that, in general, weeks to months of resistance training increases mean (type I and II) fCSA. While there have been a handful of training studies that have integrated both histological and TEM methods to describe microscopic and ultrastructural adaptations (Luthi et al., 1986; Toth et al., 2012), most of the post-1980s research in this area only performed histological assessments given that TEM methodologies are painstaking and not widely accessible. Comparatively fewer investigations have used biochemical methods to assess skeletal muscle myofibrillar and/or sarcoplasmic protein concentration adaptations following periods of training, and these studies are discussed below in greater detail. In addition to the seminal microscopy work discussed above, early post-spaceflight investigations of muscle mass and thin filament density changes from LeBlanc et al. (2000) and Riley et al. (2000) also paved the way for MRI and delicate microscopic inquiry of muscle adaptation to training. The aforementioned literature demonstrates that a common goal of several laboratories for over a century has been to assess how training affects indices representative of skeletal muscle hypertrophy. Yet, a clear definition of hypertrophy is difficult to elucidate and depends on which literature, or scientist, is consulted.

## What Are the Molecular Underpinnings of Skeletal Muscle Hypertrophy?

In 2010, a co-author of the current review (B.J.S.) defined skeletal muscle hypertrophy as an expansion of contractile elements and extracellular matrix of skeletal muscle cells (Schoenfeld, 2010), pointing to the addition of sarcomeres in parallel and/or addition of myofibrils being largely responsible for increases in cell size based on supporting evidence from Paul and Rosenthal (2002) and Tesch and Larsson (1982). Although this hypothesis is logical, neither investigation clearly provided counts of myofibrils or sarcomeres, which warrants reconsideration of the specific mode of short-term resistance training-induced hypertrophy in human fibers. Lloyd (2013) provided the elegantly simple definition of cell growth as mass accumulation. Given the potential for conflicting definitions, it seems appropriate to operationally define skeletal muscle hypertrophy as an increase in skeletal muscle mass. However, this definition may oversimplify the compositional alterations that occur during skeletal muscle hypertrophy in response to resistance training that can affect muscle function. Alternatively stated, if hypertrophy is defined as an increase in mass, then this definition insufficiently communicates *what* specifically is altered within a skeletal muscle cell in response to resistance training and *how* the increase affects muscle function. Moreover, since the type of resistance training (e.g., chronic heavy versus light loading) likely results in specific compositional alterations, this consideration is vital to the comprehensive understanding of the specificity of skeletal muscle adaptation.

As stated above, resistance training-induced hypertrophy is thought to occur primarily through the addition of sarcomeres in series or in parallel in existent myofibrils (i.e., sarcomerogenesis), or due to the synthesis of new myofibrils in existent muscle fibers (i.e., myofibrillogenesis). Paul and Rosenthal (2002) eloquently summarized this model by stating:

“… *muscle fibers may increase in diameter, as is found in singly innervated muscles, to increase the number of myofibrils in parallel. Alternatively, intrafascicularly terminating fibers could elongate, effectively increasing the number of fibers as well as myofibrils in parallel*” (p. 751).

In reference to sarcomerogenesis, Wisdom et al. (2015) posited, “On the subcellular scale, in response to elevated forces, more sarcomeres, the force-producing units of muscle, are built and added in parallel, increasing muscle cross sectional area” (p. 207). Interestingly, Wisdom and colleagues cited work from Johnson and Klueber (1991) and Farup et al. (2012) as evidence to support their hypothesis, although neither study directly assessed sarcomere abundance or myofibrillar protein concentration. Upon activation, muscle contractile force is produced by myosin and actin cross-bridge formation, the myosin power stroke, and transmission of tensile forces across the fiber (Tyska and Warshaw, 2002; Karatzaferi et al., 2004). Therefore, at the single fiber level, hypertrophy has been posited to coincide with the maintenance of or increase in specific tension (N/cm^2^) through the addition of sarcomeres in parallel (Wisdom et al., 2015; Dankel et al., 2018). However, select evidence has failed to confirm this relationship. For example, Meijer et al. (2015) have reported that *lower* specific tensions (N/cm^2^) exist in single fibers isolated from bodybuilders with significantly larger fCSAs when compared to muscle fibers from controls and power athletes. Furthermore, in 1969 (although using a surgical ablation model in rodents) Rowe reported ∼25% decreases in specific tension although CSAs of muscle samples significantly increased (Rowe, 1969). These conflicting observations could be explained by differential mechanisms of fCSA increases. For instance, fCSA and cell mass can increase through an expansion of other cell constituents in lieu of contractile protein changes (e.g., ICF, t-tubule or sarcoplasmic reticulum volume, sarcoplasmic proteins, or mitochondrial volume). Indeed, as far back as 1976 Seiden reported robust increases in fiber diameters of rodent muscle were largely associated with increases in sarcoplasmic reticulum and t-tubule volumes in the context of “work-induced hypertrophy” while no apparent increases were observed in myofibril densities (Seiden, 1976). Also, it stands to reason that some individuals may realize significant increases or decreases in myofibrillar protein content or myofibril number while others may not in response to the same training or unloading intervention. Research in humans in this regard seems equivocal (D’Antona et al., 2006; Trappe, 2009; Canepari et al., 2010; Meijer et al., 2015).

A lack of evidence explicitly showing serial or parallel sarcomere number increases in human fibers after resistance training precludes the confident conclusion that myofibrillar protein accretion is largely responsible for, or proportional to, increases in fCSA. In fact, upon extensive review of the literature, it remains to be clearly determined if increases in sarcomere or myofibril number, and therefore protein abundance, strongly correlate with increases in fCSA in response to short-term resistance training in human fibers. Conversely, and as suggested by MacDougall’s seminal 1982 publication, select evidence points to the contrary, at least in the short-term. For instance, Hody et al. (2011)^1^ used proteomics to analyze VL muscle obtained from humans prior to and following 2 weeks of eccentric-emphasis resistance training and reported a *decrease* in various contractile proteins and an *increase* in the expression of oxidative enzymes. As noted early on by Goldspink and Howells (1974), these observations could be related to the specific dose of resistance training. There are several reasons why this may be the case. For example, early adaptations to muscle fibers in response to novel training stimuli may consist primarily of preparatory structural remodeling or metabolic changes that set the stage for subsequent growth. Also, cell damage experienced in response to a novel training stimulus is associated with increased swelling/edema and this could affect assessments of protein abundance at various time points. Nevertheless, decades of anecdotal observations in the practical setting of individuals realizing significant increases in muscle mass in response to chronic resistance training suggests eventual increases in contractile protein abundance. Moreover, recent evidence from van der Pijl et al. (2018) in mice suggests increased sarcomeres in series and parallel resulting in hypertrophy in response to unilateral diaphragm denervation.

Additionally, a host of studies employing tracer methods reporting significant increases in myofibrillar protein synthesis rates in response to resistance training in the fasted and fed states provide robust support for the notion that chronic resistance training results in myofibrillar protein accretion (Louis et al., 2003; Kim et al., 2005; Moore et al., 2005; Cuthbertson et al., 2006; Carrithers et al., 2007; Kumar et al., 2009, 2012; West et al., 2009; Burd et al., 2010; Holm et al., 2010; Camera et al., 2012; Witard et al., 2014). Notwithstanding, while these tracer studies have been insightful, it is noteworthy that the measurement of myofibrillar protein synthesis rates are not synonymous to measurements of myofibrillar protein concentrations, myofibril number, or sarcomere number. Moreover, it seems the investigation of ultrastructural and biochemical alterations to resistance training have been deprioritized in lieu of delicate tracer work. Consequently, the hypothetical model of proportional increases in myofibrillar protein concentration concomitant with increases in fCSA lacks robust evidence. With this in mind, macroscopic and microscopic assessments of hypertrophy are briefly described below while a comparatively greater breadth of attention is devoted to the molecular assessment of hypertrophy in the “Molecular assessment” section that follows.

## Measurement of Hypertrophy

For parsimony, we divide measurement techniques pertaining to each of these levels into the following categories: (a) macroscopic measurements, (b) microscopic measurements, and (c) molecular measurements.

### Macroscopic Assessment

**FIGURE 3:**
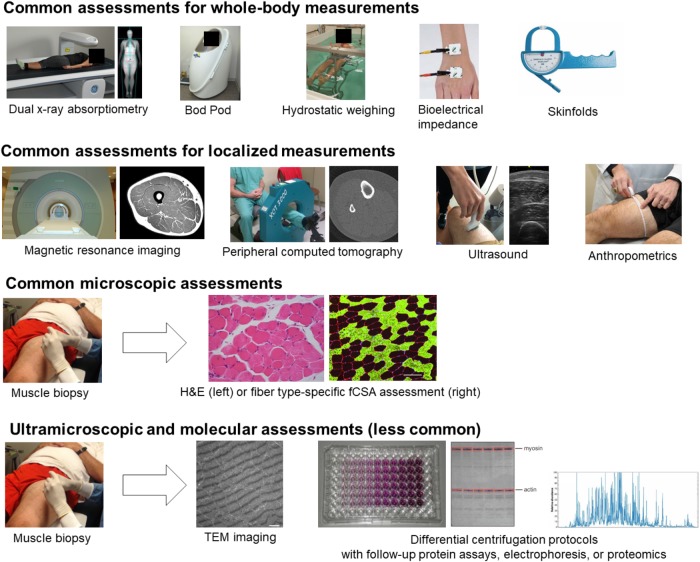
Different assessments used to monitor resistance training-induced adaptations. The diagramed techniques are utilized to measure whole-body adaptations down to molecular adaptations to resistance training. Particular attention in this review is devoted to localized, microscopic, ultramiscroscopic, and molecular assessments. Images are either from our laboratory or were obtained online where reuse for educational purposes was not restricted.

Assessments of whole-body composition change are not the focus of this review; however, certain whole-body and other anthropometric assessments are commonly employed to infer that muscle hypertrophy has occurred. These whole-body assessments will not be described or discussed in the same detail as more direct muscular assessments but deserve mention and brief description in this section given their macroscopic nature and inclusion in Figure 3. These include air displacement plethysmography (e.g., Bod Pod), hydrostatic weighing, bioelectrical impedance, skinfolds, and other anthropometrics involving handheld measuring tapes and tools. These techniques are primarily used to estimate fat and fat-free mass. Since skeletal muscle tends to occupy a large percentage of fat-free mass, an observed increase in fat-free mass based on these methods is commonly thought to indicate hypertrophy has occurred, although this is not necessarily the case given that many other factors contribute to fat-free mass (e.g., fluid).

Air displacement plethysmography uses whole-body densitometry to estimate body composition (fat and fat-free mass). Hydrostatic or underwater weighing measures mass per unit of body volume in order to calculate body density and is based on Archimedes’ principle. Once body density is calculated, fat and fat free mass can be estimated. Bioelectrical impedance estimates total body water by calculating opposition to the flow of an electric current. Fat and fat-free mass can be estimated from total body water. Skinfolds are performed by pinching specific locations of skin and underlying subcutaneous adipose tissue relative to precisely measured anatomical locations. Skinfold thicknesses can be summed and used to estimate body composition using various formulae. Technically, anthropometrics encompasses any assessment of body size, shape, and composition. However, in Figure 3, we are referring to the systematic measurement of segment girths in order ascertain changes in size potentially due to hypertrophy. While each of these assessments can be considered macroscopic, they do not directly assess muscle tissue.

The focus of this section is on common measurements in research studies which aim to directly assess muscle tissue and intuit that muscle hypertrophy has occurred. A description of each measurement approach is outlined below in order to provide context as to how each measure differs and what the implications of those differences may be. A host of measurement techniques exist to assess hypertrophy in accordance with a set dimension; that is, thickness (1D), CSA (2D), volume (3D), and mass.

#### Muscle Thickness Assessment Using Ultrasound

Muscle thickness, as assessed by B-mode ultrasonography, is a rapid, easy, relatively inexpensive, and non-invasive assessment of gross muscle size. To measure muscle thickness, investigators often image the mid-belly of a muscle and measure the linear distance between the deep and superficial aponeurosis of the muscle of interest (Franchi et al., 2018b). While muscle thickness has been shown to be highly reliable in a range of muscles (intra-class correlations, or ICCs = 0.65–0.94) (Thoirs and English, 2009), it is limited in that it is only representative of one dimension of the muscle. For example, relative to muscle thickness, muscle width, and length may hypertrophy differently, and proximal changes may be different than distal changes. This point was recently demonstrated by Vigotsky et al. (2018), who reported that hypertrophy of different regions of the same muscle are not strongly correlated within an individual. Additionally, ultrasound is highly dependent on the skill of the investigator, given that differences in the pressure exerted by the transducer against the skin can result in substantial variations in measurements and thus high inter-rater error rates. Thus, ultrasound-based assessments of muscle thickness provide a fast and practical assessment of 1D muscle size, but the quality of these assessments may very well be rater-dependent.

#### DXA

Dual-energy x-ray absorptiometry was originally designed to measure bone mineral parameters and is now a widely used method to assess skeletal muscle mass changes. Whole-body DXA scans render 2D images, although these scans can provide mass and density estimates when calibrated against a phantom of known density. Additionally, region of interest tools can be used to quantify appendicular lean masses. Newer DXA scanners have been shown to produce excellent test-retest ICCs with regard to assessing fat-free/bone-free lean tissue mass during whole-body scans (e.g., >0.99) (Kephart et al., 2016). Unlike one-dimensional ultrasound assessments and other methods discussed below, however, DXA has the inability to discriminate between muscle groups. Additionally, while DXA can distinguish bone, fat, and fat-free/bone-free lean tissue, it cannot distinguish muscle tissue from intramuscular fluid, nor is it sensitive enough to detect intramuscular fat. DXA can also be highly influenced by hydration status and other factors described by Nana et al. (2015) in greater detail which also persuade careful methods and interpretation. Notwithstanding, DXA can be used as a non-invasive assessment of gross and segmental lean body mass.

#### CT

Computed tomography was introduced in the early 1970s (Hounsfield, 1973) and has the ability to provide high-contrast, 2D images with pixel intensities related to tissue density. Tissues often measured include adipose and skeletal muscle (Heymsfield et al., 2014). When used as a measurement of muscle hypertrophy, it is common for images to be manually segmented for specific muscles or muscle groups and then quantified. CT is considered a reliable and valid method of assessing changes in muscle CSA. For example, Verdijk et al. (2009) reported coefficients of variation of 0.6% for repeated scans of muscle CSA using CT methods and inter- and intrarater reliability coefficients of 0.996 and 0.997, respectively. A drawback to CT scanning is that subjects are exposed to larger doses of radiation relative to DXA along with being costly per scan (Prado and Heymsfield, 2014).

#### pQCT

Peripheral quantitative computed tomography (pQCT) was originally developed for bone density (Gasser, 1995), but has been validated to measure training-induced changes in muscle size (DeFreitas et al., 2010) and has been reported to largely agree with MRI measurements (*R*^2^ = 0.98). Beyond the work of DeFrietas et al. to our knowledge, there are no other studies directly comparing pQCT with other measures in response to a hypertrophy stimulus pointing to the need for researchers to consider this method of assessment in future work. A strength of this measurement is the ability to detect intramuscular fat concentration which could indicate skeletal muscle quality (Sherk et al., 2014). Similar to DXA and CT, however, a limitation of the pQCT is that it cannot distinguish between muscle tissue and intramuscular fluid. Therefore, it likely reflects changes in contractile protein as well as potential alterations in training-induced fluid shifts or glycogen changes. There also seem to be no standardized protocols for imaging or analysis, so comparing studies is difficult (Erlandson et al., 2016).

#### Panoramic/EFOV Ultrasound

Panoramic and extended-field-of-view (EFOV) ultrasound are new techniques that use traditional ultrasound B-mode imaging, but “stitch” together a series of images, so as to reconstruct a larger, wider 2D image. These techniques have primarily been applied for the assessment of anatomical CSA (ACSA) and fascicle length. Research shows that panoramic ultrasound displays a range of concordance correlation coefficients (CCC) when estimating hypertrophy and atrophy compared to MRI (CCC = 0.37–0.78) (Scott et al., 2017). Axially, EFOV ultrasound has been shown to be both valid and reliable for assessing extensor carpi ulnaris fascicle lengths (Adkins et al., 2017). Additionally, mid-thigh area muscle assessments using EFOV ultrasound and CT scans have been shown to agree well with each other (Noorkoiv et al., 2010). However, Bland-Altman plots have demonstrated that panoramic ultrasound images from several muscle groups typically yield lower ACSA values compared to MRI values (Scott et al., 2012). These findings highlight the nuances required when interpreting results using whole muscle imaging, in that there are likely muscle- and dimension-specific differences in validity and reliability.

#### MRI

Magnetic resonance imaging is non-invasive, has excellent resolution, allows discrimination between separate muscles, and is commonly thought of as the reference standard for regional muscle mass assessment (Smeulders et al., 2010). MRI uses radio pulse waves to induce the nuclear spin of atomic particles – particularly those in hydrogen – and electromagnetic fields are used to localize those particles. Therefore, MRI is particularly useful for studying hydrogen-dense soft tissue, such as adipose tissue and skeletal muscle. For MRI-based volume assessments, a series of 2D cross-sectional slices are obtained and integrated as a function of distance to obtain volume (i.e., $V=\int_{orig}^{ins}ACSA\left(x\right)dx$). Test-retest values for select upper and lower-body muscles have yielded exceptionally high ICCs (e.g., 0.99) (LeBlanc et al., 2000; Smeulders et al., 2010). Although these measures are the most accurate in terms of capturing changes in gross muscle size, MRI equipment is not widely accessible and the scans are costly; therefore, its use is scarcer in the literature. Furthermore, while MRI is considered the gold standard for assessing the 2D area or segmental volume of a particular muscle group, it does not glean molecular adaptations that occur within fibers [e.g., changes in contractile protein concentration, sarcoplasmic protein concentration, intra- versus extracellular fluid (ECF), etc.] nor does it uncover the metabolic and functional nature of the tissue compared to other methods (Hellerstein and Evans, 2017).

#### Three-Dimensional Ultrasound (3DUS)

Three-dimensional ultrasound is a newer, promising approach to capturing 3D muscle architecture with standard B-mode ultrasound. By combining ultrasonography with 3D motion capture, the location and orientation of each frame of an ultrasound video can be transformed into the lab coordinate system (Mozaffari and Lee, 2017). Thus, the muscle boundaries in each digitized frame can be reconstructed in 3D, from which muscle volume (or area) can be calculated. These approaches have been validated against MRI (ICC > 0.99) (Barber et al., 2009). In addition, 3DUS allows for the quantification of fascicle-level geometry in 3D (Rana et al., 2013), which classically has been difficult to measure. Such insights show promise for improving our understanding of not only muscle geometry, but also function (Franchi et al., 2018b).

### Microscopic Assessment

Skeletal muscle hypertrophy has been assessed on the microstructural level using fCSA measurements and prepared using histochemical staining after samples are sliced and attached to microscope slides. This technique has been used to evaluate the structure and size of muscle samples since the late 1800s. Gunnar Nÿstrom was one of the first to utilize these methods in the evaluation of muscle tissue in mice by staining cardiac musculature with black India ink and using light microscopy to examine the structure of the samples. While his interests ultimately were the transverse tubules, he also found light and dark bands (isotropic and anisotropic bands, respectively) stretching the distance of each sarcomere (Nystrom, 1897). Shortly thereafter, the first study on skeletal muscle hypertrophy was conducted by Morpurgo examining the effect of run training on skeletal muscle hypertrophy in dogs which had their left sartorius muscle removed before undergoing 2 months of training (Morpurgo, 1897). After sacrificing the dogs, the right sartorius muscle was evaluated using light microscopy to examine sections adhered to slides with particular interest in fCSA change. Much of the work evaluating hypertrophy at the cellular level between the late-1800s and the mid-1900s utilized animal models, and similar to present day, the sectioning of muscle tissue for observation using microscopy. As mentioned previously, it was not until the Bergstrom needle was introduced in 1962 that these methods were utilized more openly in research related to skeletal muscle hypertrophy in humans. The Bergstrom method can yield tissue weights ranging from ∼25 to ∼300 mg.

Studies conducted prior to 1960 such as the aforementioned, and those of the modern era share many of the same fundamentals of sample extraction and preparation with many of the differences coming via technological advancement of imaging programs and the introduction of computer based models to aid in evaluation. The procurement of muscle biopsies are safe, minimally invasive, and can be performed as an outpatient procedure using the modified Bergstrom technique (Shanely et al., 2014). The basic methods of tissue processing are as follows (Verdijk et al., 2009; Mobley et al., 2017): (a) approximately 20–40 mg of tissue is typically placed into a cryomold with media before slow freezing in isopentane cooled by liquid nitrogen and subsequent storage at -80°C, (b) samples are cut in thin slices ranging ∼5–20 μm thick and are adhered to a slide, and (c) different methods can be used to stain the samples for evaluation of mean fCSA or fiber type-specific fCSA such as those described in Haun et al. (2017), Roberts B.M. et al. (2018), Wang et al. (2015), and Verdijk et al. (2009). In general, light is emitted from the microscope and passed through a filter to isolate a specific frequency which is absorbed by the specimen, and nanoseconds later, light is returned from the specimen (Stokes shift) which is filtered by selected band pass filters (FITC, DAPI, TRITC, etc.) to illuminate the image returned in a certain manner based on the fluorophores used in the staining process (Sanderson et al., 2014). Typically, the returned images are captured between 10 and 20× magnifications before being evaluated through specialized software or manual measurement, although different laboratories employ distinct techniques which have contributed to some of the variation in findings (further discussed below).

The hypertrophic response to resistance training correlates strongly to the volume of training as discussed by Schoenfeld (2010), suggesting a dose response relationship exists. In this regard, Tesch and Larsson (1982) found that muscle tissue from the medial deltoid and the VL obtained from high level bodybuilders exhibit larger slow twitch fiber fCSA values compared to recreationally trained individuals, although fibers exhibiting fast twitch properties yielded similar fCSA values between cohorts. Additionally, Meijer et al. (2015) reported that bodybuilders completing moderate to high volume training display greater mean fCSAs compared to untrained individuals and strength/power athletes. However, it is also important to note that while all fiber types were larger, there was a significant difference between type I fCSA of bodybuilders as compared to untrained individuals and strength/power athletes, yet no meaningful difference between type I fCSA of the untrained group and the strength/power group. Additionally, Eriksson et al. (2006) conducted a study in high level powerlifters, some of which self-reported use of anabolic steroids, and found that there may be potential for fiber splitting along the length of a muscle fiber with chronic high intensity loading which could have the potential to present an increase in fiber quantity and perhaps a decrease in mean fCSA when analyzing via microscopy.

Interestingly, fCSA adaptations to resistance training are usually relatively greater than any other hypertrophy surrogates when expressed as percent change (described in the “Measurement Agreement” section). Regarding fCSA hypertrophy, consideration of the fiber type (e.g., I, IIa, and IIx) seems warranted, as evidence has revealed fibers exhibit different magnitudes of hypertrophy following periods of resistance training (Fry, 2004). Reporting of mean fCSA, which accounts for all fibers, may result in different change scores compared to reporting type I or type IIa muscle fibers independently. Kosek et al. (2006) reported that mean type II fCSA (which weights the percent distribution of both IIa and IIx fibers) exhibited ∼32% hypertrophy in young adult fibers following 16 weeks of resistance training, yet type IIa fibers demonstrated ∼25% hypertrophy and type I fibers ∼18%.

Although a sensitive assessment of skeletal muscle hypertrophy, there are also limitations of fCSA calculations, such as the method of tissue processing, biopsy location, and measurement methods. For example, it is practically impossible to biopsy the same location in a muscle twice, so any changes in size observed are assumed to extrapolate to surrounding fibers, or the same fibers along their length. The climate of a laboratory, buffers, and other factors during tissue processing can affect measurements of fiber size. For instance, since a muscle cell is ∼70% fluid, tissue processing can create variability of water retention in the muscle sample; and this could potentially alter findings assuming unstandardized processing procedures from one sample or from one laboratory to the next. Muscle glycogen concentration could also affect muscle fCSA, and this variable often remains unreported in manuscripts providing fCSA measurements. Additionally, muscles can hypertrophy in a non-uniform manner (distal versus proximal), which would not be detected with single-site measurements (Narici et al., 1996). Furthermore, a variety of fiber-sizing software and laboratory methods described in the published literature could differentially affect percent change calculations. It is common for authors to devote only a few sentences to description of how fibers are sized upon image capturing. For example, some laboratories randomly select 25–50 fibers for manual sizing while others size 100 or more using specialized software (Lau et al., 2018; Wen et al., 2018). Unfortunately, this often leaves absent specific calibration procedures, disclosure of the training protocol used for the rater, and the specific software or technique used to calculate fCSAs. In this regard, it has been reported that VL type II fCSA increases from ∼6,000 to ∼8,400 μm^2^ in college-aged men following 12 weeks of resistance training (Snijders et al., 2016), whereas our laboratory as well as Reidy et al. have reported that VL type II fCSA increases from ∼5,100–5,500 to ∼6,000–6,500 μm^2^ during this same time course in this same population (Mobley et al., 2017; Reidy et al., 2017b). Another independent laboratory has reported that VL type II fCSA increases from ∼6,200 to 7,500 μm^2^ in college-aged men following 16 weeks of resistance training (Bellamy et al., 2014). Considering these apparent discrepancies, it stands to reason that research subject characteristics like previous or current activity levels and nutrition habits could be contributing factors; while differences in the training interventions and tissue processing methods between laboratories could also explain the disagreement. We feel that both more appropriate and transparent method descriptions and standardization between labs can help resolve some of these discrepancies moving forward.

### Molecular Assessment

The molecular signature that coincides with skeletal muscle hypertrophy in response to resistance training has largely been understudied, but can be inferred by analyzing changes in protein sub-fractions within biopsied tissue through differential centrifugation protocols followed by simple biochemical assays (e.g., Bradford or bicinchoninic acid assays), polyacrylamide gel electrophoresis, immunoblotting for specific proteins of interest, or larger-scale proteomic-based assessments. Although theoretically simple, these methods produce practical challenges that often prevent laboratories from employing them. However, we feel this area of inquiry is vital to our comprehensive understanding of skeletal muscle adaptation to training. The measurement of skeletal muscle myofibrils dates back at least as far as 1957 when Hanson and Huxley (1957) used extraction, solubilization, and polyacrylamide-gel based separation techniques to quantify myosin and actin concentrations. More recent studies have used various methods to determine how resistance training affects myofibrillar and/or sarcoplasmic protein concentrations, and given that assessment methods have been inconsistent, discordant findings have resulted. For instance, Shelmadine et al. (2009) reported ∼50% increases in myofibrillar protein concentration following 28 days of resistance training. Willoughby and Rosene (2001) similarly reported ∼40% increases in myofibrillar protein concentration after 12 weeks of resistance training, and have also reported ∼85% increases in myofibrillar protein concentrations 6 h after a single session of resistance training (Willoughby and Nelson, 2002). Cribb and Hayes (2006) and Cribb et al. (2007) have reported similar increases in myofibrillar protein concentration after 10 weeks of resistance training in two separate studies. However, a comparatively greater number of authors have reported no alteration in protein concentration or an apparent decrease in response to resistance training. For instance, Brook et al. (2015) reported no significant change in total soluble protein concentration after 6 weeks of resistance training (pre: 521 ± 34 mg/g, post: 552 ± 28 mg/g dry weight). Haus et al. (2007) reported no significant change in myofibrillar, sarcoplasmic, myosin, or actin protein concentration after either 35 or 90 days of resistance training 2–3 days per week. Woolstenhulme et al. (2006) reported no significant change in protein concentration after 8 weeks of resistance training although significant increases in type II fCSA occurred. Carrithers et al. (2002) reported no significant change in total, sarcoplasmic, myofibrillar, myosin, or actin protein concentrations after 5 weeks of resistance training. Trappe et al. (2011) reported no significant change in the total percentage of water or protein content of biopsy samples after 12 weeks of resistance training in older adults. Our laboratory recently reported that high and low hypertrophic responders to 12 weeks of resistance training did not (on average) present increases in myofibrillar or sarcoplasmic protein concentrations (Roberts M. D. et al., 2018). Although assessed through TEM, it is notable that Toth et al. (2012) and MacDougall et al. (1982) have both reported decreases in myofibril density concomitantly occur with fCSA increases following 18 weeks and 6 months of resistance training, respectively, although Luthi et al. (1986) reported no change in myofibril density following 6 weeks of training. Additionally, the aforementioned work by Penman in the 1960s suggest reductions in myosin density occur following brief periods of resistance training (Penman, 1969). Although in animal muscle samples, other authors have also reported no change or reductions in myofibril density in response to short-term resistance training-induced hypertrophy (Goldspink and Howells, 1974; Seiden, 1976). Furthermore, the studies described previously showing lower *specific* tension (i.e., N/μm^2^) in larger fibers or hypertrophied fibers in response to resistance training seem to support a potential dilution of myofibrils. A summary of these human studies are presented in Table 1.

**Table 1:** Human studies observing ultrastructural changes from muscle biopsy specimens with resistance training.

Author/Year	Subject and group description	Methods	Outcomes
Penman, 1969	College age males	(a) Eight weeks intervention	Myosin density: ↓ in all groups Actin and myosin filament diameters: ↑ in all groups
	(a) Isotonic leg extension training (*n* = 2)	(b) VL biopsies obtained prior to and following the intervention	
	(b) Isometric leg extension training (*n* = 2)	(c) TEM examination of myosin fiber density, distance between myofilaments, myosin filament diameter, actin filament diameter	
	(c) Run training (*n* = 2)		

Penman, 1970	College age males	(a) Ten weeks intervention	fCSA: ↔ Distance between myosin filaments: ↓
	(a) Leg RT and running (*n* = 3)	(b) VL biopsies obtained prior to and following the intervention	
		(c) TEM examination of myosin fiber density, distance between myofilaments, myosin filament diameter, actin filament diameter	

MacDougall et al., 1982	Untrained males (UT) and well trained bodybuilders and powerlifters (WT).	(a) Histological methods for biceps brachii fCSA	Type I fCSA: UT-pre = UT-post = WT
	(a) UT (*n* = 5)	(b) TEM for MF, SARCO and MITO areas	Type II fCSA: UT-pre < UT-post = WT
	(b) WT (*n* = 7) (WT average training age = 7 years)		MF area: UT-pre > UT-post > WT
			SARCO area: UT-pre < UT-post < WT
			MITO area: UT-pre > UT-post = WT

Luthi et al., 1986	Untrained males (*n* = 5)	(a) Six weeks intervention, 3 days/week	VL CSA (CT): ↑ fCSA: ↔
		(b) CT scan for CL CSA	MF area: ↔
		(c) Histology for VL fCSA	MITO area: ↔
		(d) TEM for MF and MITO areas	

Willoughby and Rosene, 2001	Untrained males	(a) Twelve weeks intervention. Whole body RT 3 days/week	MF protein concentration: ↑ in both groups
	(a) Supplemental creatine (*n* = 8)	(b) Biochemical assays used for VL MF protein which was isolated using TRIzol-based methods (no histology)	
	(b) Placebo (*n* = 8)		

Carrithers et al., 2002	Untrained males and females	(a) VL and soleus biopsies completed prior to and following 5-week intervention	VL total protein: ↔ in any group
	(a) Unilateral Limb Suspension (ULLS) (*n* = 11)	(b) Biochemical assays for either total protein, cytosolic protein, myofibrillar protein, myosin concentration, and actin concentration	VL cytosolic protein: ↔ in any group
	(b) Resistance training (RT) (*n* = 10)		VL MF protein: ↔ in any group
	(c) ULLS+RT (*n* = 10)		VL myosin concentration: ↔ in any group
			VL actin concentration: ↔ in any group
			Other notes: total protein, cytosolic protein and MF protein decreases were observed in the soleus muscle of the ULLS group

Cribb and Hayes, 2006	Trained males	(a) Ten weeks whole body RT, 3 days/week	fCSA (type I and II): ↑ in both groups
	(a) PRE/POST (*n* = 8)	(b) VL biopsies prior to and following the 10-week intervention	MF concentration: ↑ in both groups
	(b) MORN/EVE (*n* = 9)	(c) Histological methods for VL fCSA	
		(d) Biochemical assays used for MF protein	

Cribb et al., 2007	Trained males (*n* = 31)	(a) Ten weeks whole body RT, 3 days/week	fCSA (type I and II): ↑ in all groups,
	(a) PRO (*n* = 10)	(b) VL biopsies prior to and following the 10-week intervention	Cr-PRO-CHO greater type II increase after 10 weeks
	(b) PRO-CHO (*n* = 11)	(c) Histological methods for VL fCSA	MF concentration: ↑ in all groups,
	(c) Cr-PRO-CHO (*n* = 10)	(d) Biochemical assays used for MF protein	Cr-PRO-CHO greater increase after 10 weeks

Woolstenhulme et al., 2006	Untrained males	(a) Untrained males performed either 8 weeks lower body RT 3 days/week or single bout of RT	Type I fCSA: no difference between trained and single-bout
	(a) Single bout (*n* = 6)	(b) VL biopsies prior to and following 8-week intervention	Type II fCSA: ↑ in trained, but ↔ in single bout
	(b) Eight weeks RT (*n* = 6)	(c) Biochemical methods for total protein concentration	Total muscle protein concentration: ↔ in either group desmin protein concentration: ↑ in trained, but ↔ in single bout actin protein concentration: ↔ in either group
		(d) Histology for fCSA	Dystrophin protein concentration: ↔ in either group
		(e) Immunoblotting for desmin, actin, and dystrophin	

Haus et al., 2007	Untrained males	(a) Subjects completed either 35 days ULLS, 35 days ULLS+RT, 90 days BR, 90 days BR+RT.	35 days ULLS: ↓ in muscle volume
	(a) Unilateral Limb Suspension (ULLS) (*n* = 11)	(b) Assessments prior to and following either 35 or 90 days which included VL muscle biopsies and mid-thigh MRI	35 days ULLS+RT: ↑ in muscle volume
	(b) ULLS+RT (*n* = 10)	(c) Biochemical methods for assessment of muscle protein quantification as mixed, sarcoplasmic, and myofibrillar	90 days BR: ↓ in muscle volume
	(c) Bed rest (BR) (*n* = 9)	(d) SDS–PAGE methods for assessment of myosin, actin, and collagen protein concentrations	90 days BR+RT: ↔ in muscle volume
	(d) BR+RT (*n* = 8)		All groups: ↔ mixed protein, SARCO protein, MF protein, myosin, actin, or collagen protein concentrations

Shelmadine et al., 2009	Untrained males	(a) Four weeks intervention, 2 days/week upper and 2 days/week lower split	MF concentration: ↑ in both groups
	(a) Supplemental pre-workout (*n* = 9)	(b) Biochemical assays used for VL MF protein which was isolated using TRIzol-based methods (no histology)	
	(b) Supplemental placebo (*n* = 9)		

Trappe et al., 2011	Older adults (*n* = 36)	(a) Twelve weeks knee extensor resistance exercise	Quadricep muscle volume: ↑ in all groups, acetaminophen and ibuprofen ↑ more than placebo
	(a) placebo (*n* = 12)	(b) MRI measurement of quadricep muscle volume	Muscle protein content: ↔ in all groups
	(b) acetaminophen (*n* = 11)	(c) VL biopsy prior to and following 12 weeks intervention	Muscle water content: ↔ in all groups
	(c) ibuprofen (*n* = 13)	(d) Biochemical assays used for muscle protein and water content (% muscle wet weight)	

Toth et al., 2012	(a) Heart failure patients (HFP) (*n* = 10)	(a) Eighteen weeks whole body RT	fCSA (type I and II): ↔ in both groups
	(b) Minimally active people (CTL) (*n* = 14)	(b) VL biopsies prior to and following 18-week intervention	MF area: ↓ in both groups
		(c) Single muscle fiber morphology (cross-sectional area)	A-band length: ↑ in both groups
		(d) Electron-microscopy-based ultrastructural measurements	
		(e) Single fiber mechanical measurements.	

Brook et al., 2015	Untrained males (*n* = 10)	(a) Assessments prior to and following 6 weeks of unilateral leg RT (noted as T), with contralateral leg serving as control (noted as UT)	Thigh lean mass: ↑ in T but not UT
		(b) VL biopsies prior to, middle, and following the intervention	VL thickness: ↑ in T but not UT
		(c) Mid-thigh muscle architecture and DXA-derived mass	VL fiber length and pennation angle: ↑ in T but not UT
		(d) VL myofibrillar fractional synthesis rate	Myofibrillar FSR: ↑ in T but not UT
		(e) VL total protein, DNA, RNA concentrations using spectrophotometry	VL total protein: no difference between T and UT

Reidy et al., 2017a	Untrained males (*n* = 31)	(a) Twelve weeks whole body RT	fCSA: ↑
		(b) VL biopsies prior to and following 12-week intervention	Post-absorptive MPS: ↑
		(c) Histological methods for VL fCSA	Post-absorptive MPB: ↓
		(d) Post-absorptive MPS and MPB assessments	Muscle protein concentration: ↔
		(e) Biochemical assays for muscle protein concentration, DNA concentration, water content	Muscle water content: ↑
		(f) VL Ultrasound, MRI, and DXA	VL thickness: ↑
			Leg volume: ↑
			Leg lean mass: ↑

Roberts M. D. et al., 2018	Untrained males	(a) Twelve weeks whole body RT	fCSA (type I and II): ↑ in HI, ↔ in LO
	(a) High hypertrophic responders (HI) (*n* = 13)	(b) VL biopsies prior to and following 12-week intervention	MF concentration: ↔ in HI, ↔ in LO
	(b) Low hypertrophic responders (LO) (*n* = 12)	(c) Histological methods for VL fCSA	Myosin and actin concentration: ↔ in HI, ↔ in LO
	Assessed using combined metrics.	(d) Biochemical assays used for MF protein, SARCO protein and MITO volume	SARCO concentration: ↔ in HI, ↔ in LO
		(e) SDS–PAGE for actin and myosin content	MITO content: ↔ in HI, ↔ in LO

Unpublished data from Haun et al., 2018	Previously trained college age males; only high hypertrophic responders (HI) represented (*n* = 15)	(a) Six weeks whole body high volume training 3 days/weeks	fCSA (type I and II): ↑
	Response determined VL mean fCSA increases	(b) VL biopsies prior to, middle, and following the intervention	Myosin and actin concentration: ↓
		(c) Histological methods for VL fCSA	Phalloidin staining intensity/fiber: ↓
		(d) Biochemical assays used for SARCO protein and MITO volume	SARCO protein concentration: ↑ (*p* = 0.065)
		(e) SDS–PAGE for actin and myosin content	MITO content: ↓
		(f) Phalloidin staining for contractile protein content per fiber	


*These studies used transmission electron microscopy (TEM) or biochemical techniques to assess changes in myofibrillar protein content in muscle biopsy specimens following weeks to months of resistance training (RT). Bold-faced text indicates significant effects were noted. Symbols and other abbreviations: Δ, pre-study versus post-study value; ↑, significant increase; ↓, significant decrease; ↔, no change from a statistical standpoint; MF, myofibrillar fraction; SARCO, sarcoplasmic fraction; VL, vastus lateralis; fCSA, fiber cross-sectional area; SDS–PAGE, polyacrylamide gel electrophoresis.*

In interpreting these data, increases in myofiber size with no change in myofibrillar protein concentration would indicate that cellular growth occurs with proportional increases in myofibril protein accretion. That is, the stoichiometry of the muscle cell would be mostly preserved in this case. Conversely, increases in myofiber size with a decrement in myofibrillar protein concentration would indicate that the protein pool is being diluted through increases in ICF or other sarcoplasmic constituents. As mentioned above, select reports have curiously suggested that resistance training robustly increases myofibrillar protein concentrations, which would indicate that substantial myofibrillar packing occurs within the first few weeks to months of training. Alternatively stated, such findings suggest myofibril protein accretion far outpaces cell growth. Therefore, these inconsistent reports on the molecular and ultrastructural adaptations to resistance training warrant additional research.

Beyond alterations in contractile and sarcoplasmic proteins, potential fluid shifts are a commonly underappreciated molecular aspect of skeletal muscle adaptation to resistance training. Sensitive assessment of skeletal muscle fiber fluid volume has traditionally been completed by weighing frozen, hydrated samples on laboratory scales with ≤0.1 mg sensitivity, freeze-drying samples in a vacuum-sealed benchtop apparatus, and calculating the difference between hydrated and dehydrated tissue weights after re-weighing on the same scale. Only a few investigations in humans have reported alterations in wet and dry weights after a period of exercise training. For instance, Reidy et al. (2017a) reported significant increases in muscle tissue fluid content and fCSA after 12 weeks of resistance training but no significant change in protein concentration. Mora-Rodriguez et al. (2016) also reported significant increases in skeletal muscle sample water content but significant decreases in total protein concentration after 4 months of aerobic exercise training. Harber et al. (2009) similarly reported significant increases in muscle water content and significant decreases in myofibrillar protein concentrations after 12 weeks of aerobic exercise training, although fCSA measurements significantly increased. Given the potential influence fluid volume may exert on fCSA measurements used to surmise hypertrophy after an exercise intervention, it seems critical that water content be accounted for when analyzing microscopic and molecular-level hypertrophy. However, it should be noted that the freeze-drying method cannot delineate ICF from ECF levels. Thus, other techniques are needed to determine these metrics, and this is discussed in greater detail below.

## Measurement Agreement

While the direct comparison of different methods of hypertrophic assessment are sparse, there are data suggesting that macroscopic, microscopic, ultramicroscopic and/or biochemical indices of skeletal muscle hypertrophy following resistance training have poor agreement. A recent study that perhaps best demonstrates this phenomenon is by Franchi et al. (2018a), who observed that, while percent change increases in ultrasound-assessed VL muscle thickness and MRI-derived CSA showed strong correlations following 12 weeks of leg extensor resistance training (*r* = 0.69), ultrasound findings were poorly associated with MRI-derived calculations of muscle volume (*r* = 0.33). DXA is highly correlated with both MRI and CT measures of muscle area when assessed at a single point in time (Levine et al., 2000; Maden-Wilkinson et al., 2013). However, DXA shows only a moderate correlation with CT (*r* = 0.52) when assessing changes in muscle mass following regimented resistance training, and its high measurement error raises questions as to suitability for determining subtle changes in muscle mass over the course of a regimented resistance training protocol (Delmonico et al., 2008). Consistent with this hypothesis, Snijders et al. (2015) reported statistically greater increases in MRI-assessed thigh muscle CSA in a protein-supplemented versus placebo group following a 12-week resistance training program; however, lower extremity lean mass as measured by DXA failed to show statistically significant changes from pre- to post-study.

There are also differences when comparing the degree of hypertrophy between MRI and biopsy fCSA metrics. For example, Narici et al. (1996) reported a 2% increase in VL fCSA, but a 7% increase in VL CSA measured by MRI following 6 months of resistance training. These authors reported that differences existed when comparing either of those two measurements to whole quadriceps measurements, which increased by 15%. Another study by Aagaard et al. (2001) compared changes in VL muscle size using MRI, biopsy, and ultrasound after 14 weeks of resistance training. These authors reported a ∼16% increase in fCSA, yet only a ∼10% increase in muscle volume. Additionally, a positive relationship existed between the change in fCSA and CSA assessed via MRI, although this correlation was only moderate (*r* = 0.58). As previously mentioned, several studies have also noted fCSA increases in response to resistance training yield greater relative change scores compared to other hypertrophy surrogates. For example, Esmarck et al. (2001) reported a 7% increase in VL CSA assessed by MRI and a 22% increase in VL fCSA following 12 weeks of resistance training. Verdijk et al. (2009) reported an 8.5% increase in quadriceps CSA assessed by CT, but a 28% increase in Type II fCSA. Frontera et al. (1988) reported a 10% increase in quadriceps CSA assessed by CT, but ∼28% increase in VL fCSA. Moreover, when analyzing data from a recently published study from our laboratory (Haun et al., 2018), the top 10 hypertrophic responders to 6 weeks of high-volume resistance training experienced a 23% increase in right VL fCSA, although upper right lower extremity lean mass assessed by DXA only increased by 8.8% and mid-thigh thickness assessed via ultrasound only increased by 2.1%.

When associating ultrastructural and histological adaptations to resistance training, there is no clear relationship. As stated above, the landmark TEM work by Penman and MacDougall suggests that the concomitant dilution of myofibrillar proteins with fCSA increases may occur with resistance training. Remarkably, these data agree with studies performed decades later. For instance, Toth et al. (2012) reported that 18 weeks of resistance training resulted in a statistically significant 13% decrease in VL myofibrillar protein area (assessed via TEM), although no changes were noted in fCSA. Additionally, Harber’s group reported 12 weeks of cycle ergometer training in older, untrained subjects increased fCSA by ∼20%, increased quadriceps CSA (assessed via MRS) by 12%, and increased knee extensor power by 55% (Harber et al., 2009). Yet, myofibril protein concentrations (assessed through biochemical methods) *decreased* by ∼14%. Yet, Harber’s group has also shown that elite runners myofibrillar protein concentrations are higher than recreational runners (Reidy et al., 2014). Observations of increased micro-/macro-structural variables indicative of skeletal muscle growth along with improvements in functional performance metrics led the authors to justifiably conclude aerobic exercise training resulted in significant muscle hypertrophy. Nevertheless, it is intriguing that this study similarly observed various indices of muscle hypertrophy occurred in lieu of decrements in myofibrillar protein levels.

Importantly, many of the supposedly discrepant findings compared above originate from different methods and this will be further discussed in a later section. However, the aforementioned studies in this section demonstrating that different surrogates of hypertrophy seemingly disagree with each other prompted our laboratory to examine VL myofibrillar protein concentration differences between high versus low anabolic responders following a 12-week full body resistance training program (Roberts M. D. et al., 2018). Response clusters were generated based on pre- to post-training changes in right VL muscle fCSA (type I + type II fibers), VL thickness assessed via ultrasound, and total body muscle mass (TBMM) assessed via DXA. Participants in the upper and lower 25th percentiles were classified as high (HI, *n* = 13) and low (LO, *n* = 12) hypertrophic responders, respectively. Notably, this clustering method indicated HI responders presented significantly greater increases in lower body strength [assessed via three repetition maximum (3RM) back squat] relative to LO responders (mean ± SE: Δ3RM squat = +42 ± 3 kg, LO Δ3RM squat = +31 ± 9 kg; *p* = 0.005), and we noted an advantage of our clustering method included its association with a functional strength outcome. On average, pre-to-post training changes in VL fCSA were +1426 ± 253 μm^2^ (mean ± SE) in HI responders and +5 ± 209 μm^2^ in LO responders (*p* < 0.001). However, no significant between- or within-cluster changes in myofibrillar protein concentrations were observed, and regardless of response cluster, there was not an association between pre-to-post training changes in fCSA and myofibrillar protein concentrations (*r* = -0.014, *p* = 0.947). Table 2 displays Pearson’s r correlation values between whole-body, regional, microscopic, and ultrastructural indices of skeletal muscle hypertrophy from the subjects of this study.

**Table 2:** Associations between macro-, micro-, and ultrastructural surrogates of hypertrophy following 12 weeks of resistance training.

	Δ DXA TBMM (%)	Δ VL thick (%)	Δ mean fCSA (%)	Δ MF protein (%)	Δ SARCO protein (%)	Δ myosin protein (%)
Mean values (SD) (*n* = 25)	+5.9 (3.6)	+17.5 (11.6)	+17.6 (23.5)	+3.6 (39.5)	+6.8 (22.9)	+10.5 (44.7)
*p*-value relative to PRE	**<0.001**	**<0.001**	**0.002**	0.660	0.284	0.685
correlation *r*-values						
Δ DXA TBMM (%)	-	0.47	**0.54**	-0.08	-0.39	-0.20
Δ VL thick (%)	0.47	-	0.31	0.37	-0.10	0.05
Δ fCSA (%)	**0.54**	0.31	-	0.00	-0.04	0.21
Δ MF protein (%)	-0.08	0.37	0.00	-	0.45	**0.61**
Δ SARCO protein (%)	-0.39	-0.10	-0.04	0.45	-	**0.71**
Δ myosin protein (%)	-0.20	0.05	0.21	**0.61**	**0.71**	-


*These data from our laboratory (Roberts M. D. et al., 2018) are pre- versus post-training percent change scores from 25 untrained, college-aged male participants that engaged in 12 weeks of structured resistance training. Symbol and abbreviations: Δ, pre- to post-training percent change; SD, standard deviation; DXA TBMM, dual x-ray absorptiometry total body muscle mass; VL thick, ultrasound-assessed vastus lateralis thickness; fCSA, muscle fiber cross-sectional area; MF protein, myofibrillar protein assessed through biochemical methods; SARCO protein, sarcoplasmic protein assessed through biochemical methods. Other notes: myosin protein was assessed through electrophoretic methods with relative quantification being calculated through band densitometry. Findings: DXA TBMM, VL thick and mean fCSA metrics all indicated that macro- and microscopic indices of skeletal muscle hypertrophy occurred. However, no strong correlations (i.e., *r*-values > 0.80) were observed between percent change scores for these metrics. No significant changes in MF protein, SARCO protein or myosin protein occurred indicating that (on average) myofibrillar packing or dilution did not occur. There were moderate correlations (*r*-values > 0.50) between Δ MF protein and Δ myosin protein as well as Δ myosin protein and Δ SARCO protein indicating that biochemical assessments generally were better associated with each other relative to macro- and microscopic assessments. All moderate correlations are bold-faced.*

Notably, the data in Table 2 includes percent change scores in DXA TBMM, VL thickness, VL fCSA, VL myofibrillar protein concentrations, VL sarcoplasmic protein concentrations, and VL myosin protein concentrations. It is apparent that ultrastructural indices of skeletal muscle hypertrophy typically agree well with one another. For instance, moderate correlations exist for raw delta as well as percent change scores in myosin protein and myofibrillar protein concentrations (*r* = 0.61). Moderate correlations also exist for raw delta as well as percent change scores in myosin protein and sarcoplasmic protein concentrations (*r* = 0.71). However, microscopic assessments do not agree well with ultrastructural assessments or macroscopic assessments, and vice versa. To this point, we also performed simple correlations for each of the surrogate measures of hypertrophy on data from all subjects regardless of cluster for greater statistical power (aside from myofibrillar and sarcoplasmic protein concentration changes due to lack of available sample from this study). Briefly, changes in VL thickness produced correlation coefficients ranging from *r* = 0.06–0.25 in relation to changes in fCSA and DXA TBMM. Changes in type I and type II fCSA correlated weakly with changes in DXA TBMM (*r* = 0.18, *r* = 0.13, respectively).

**FIGURE 4:**
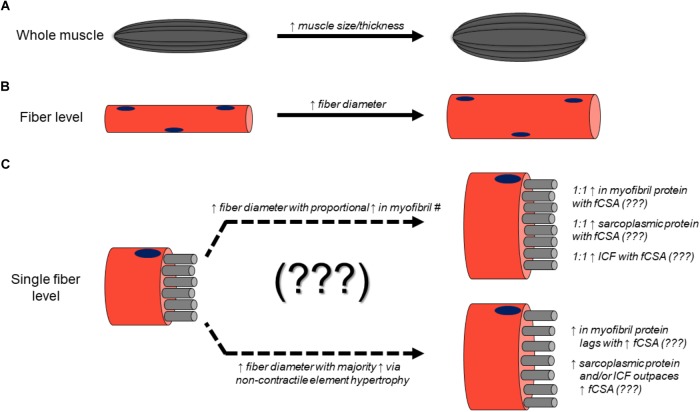
Mechanisms of resistance training-induced skeletal muscle hypertrophy. Numerous studies have demonstrated that resistance training increases muscle thickness (assessed using B-mode ultrasound) as well as muscle CSA (assessed with CT or MRI) **(A)**. Likewise, numerous studies have reported that fCSA increases occur with resistance training **(B)**. However, the ultrastructural and molecular adaptations to resistance training remain largely unresolved **(C)**.

Considering these findings against the background of the current hypothetical model of RT-induced skeletal muscle hypertrophy presents a conundrum. For decades, it has been assumed that the deposition of sarcomeres in parallel in existent myofibrils, or the genesis of new myofibrils in existent muscle fibers results in the observed expansion of fCSA and macroscopic assessments of muscle size in response to RT interventions. However, it is clear from the above data that this assumption lacks consistent and explicit empirical support. After performing an extensive search of the scientific literature, it is apparent that no studies have directly quantified sarcomere number in parallel prior to or following resistance training in human fibers. Furthermore, the few studies employing TEM methods to provide myofibril densities were severely underpowered having analyzed few fibers and few subjects, and although critical first steps, do not allow confident population-wide inferences. Strikingly, of the available evidence surveyed wherein molecular and microscopic measurements occurred prior to and following resistance training, a reduction in myofibrillar protein concentrations concomitant to observed increases in fCSA has been a more common finding. According to the widely assumed model of resistance training-induced hypertrophy, a maintenance of myofibrillar protein concentration should coincide with an increase in fCSA. Moreover, this occurrence should produce an increase in macroscopic measures of muscle size including muscle thickness, muscle mass, and muscle volume. Yet, the current state of the evidence tells a different and relatively inconsistent story. Based upon the current available evidence, Figure 4 summarizes how resistance training-induced skeletal muscle hypertrophy occurs at the macroscopic level and may occur at the ultrastructural level.

## Potential Explanations and Limitations

The first and most obvious drawback when comparing biopsy data to regional or whole body metrics is that the biopsy specimen is small (e.g., 100 mg) relative to the VL muscle, and more so, relative to the musculature of the whole body. In relation to characterizing ultrastructural indices using TEM, inadequate sampling is an imminent concern given that dozens (not hundreds) of partial fiber areas are typically analyzed (Alway et al., 1988). Another viable concern with small samples from both fCSA and TEM is a regression to the mean phenomenon from sampling sites, since it is impossible to biopsy the same location twice. This can make repeated measures difficult to correlate because large or small values can skew results (Barnett et al., 2005). Further compounding this issue is the lack of true controls in most training studies to help correct for repeated measures sampling complexity.

With regard to biochemical assays to determine myofibrillar, sarcoplasmic, myosin, and actin protein concentrations, we recently reported that an adopted method from Alfred Goldberg’s laboratory (Cohen et al., 2009) yields good separation of contractile and non-contractile proteins and is sensitive to detect 5–25% changes in protein concentrations (Roberts M. D. et al., 2018). However, it is currently difficult to decipher how resistance training affects subcellular protein concentrations given that multiple methods have been used (Willoughby et al., 2002; Cribb and Hayes, 2006; Shelmadine et al., 2009; Roberts M. D. et al., 2018), and some of these reports using TRIzol-based methods have likely not accurately reported myofibrillar protein levels given that this method (Kopec et al., 2017): (a) leads to inefficient protein retrieval due to poor solubilization of precipitated protein pellets, and (b) does not contain a sufficient detergent (e.g., Triton) to lyse membrane structures. In addition to these points, although mitochondrial volume has been consistently shown to decrease in response to resistance training (Groennebaek and Vissing, 2017), alterations in sarcoplasmic reticulum and t-tubule volumes have been largely unexplored in human skeletal muscle in response to training interventions; and changes in these structures could contribute to observed increases or decreases in fCSA. Moreover, disproportionate increases in ICF volume may occur with resistance training, albeit there is no current microscopic or biochemical method to directly decipher this phenomenon.

Limitations to various muscle imaging techniques also exist. For instance, although MRI can estimate total fluid content within a scan (Ogino et al., 1994), standard MRI, x-ray, and ultrasound measurements cannot account for potential ECF and ICF shifts that may occur with training. This is a critical point that is oftentimes underappreciated given that ECF, which does not represent the intracellular milieu and contributes to mass and thickness changes, significantly increases with higher-volume resistance training (Haun et al., 2018). Thus, the tandem use of regional bioelectrical impedance spectroscopy (BIS) with regional (e.g., thigh muscle area or volume) or whole body scans is a fruitful area for exploration. In this regard, we recently used DXA to determine that 6 weeks of voluminous resistance training increased whole-body DXA LBM by +1.34 kg from weeks 1 to 3 and an additional +0.85 kg from weeks 4 to 6 in 30 previously trained college aged males (Haun et al., 2018). When adjusting pre- to post-training changes in DXA LBM for BIS-extrapolated ECF changes, however, a significant increase occurred from weeks 1 to 3 (+1.18 kg), but not from weeks 4–6 (+0.25 kg). Hence, using methods that accurately account for ICF and ECF shifts which occur during resistance training may provide more insight as to whether true hypertrophy occurs. Although BIS-based calculations of ECF and ICF have been shown to strongly agree with sodium bromide dilution and deuterium oxide-based assessments (Moon et al., 2008; Birzniece et al., 2015), it remains to be investigated how valid and reliable BIS-based assessments are compared to other methods in context of resistance training-induced hypertrophy and fluid alterations. Furthermore, Reidy et al. (2017a) have reported ∼4 % increases in plasma volume after 12 weeks of resistance training and a significant decrease in the percentage of muscle water (-0.1–1.3%) which also persuades the measurement of whole-body and muscle biopsy sample fluid content for more accurate inference. A relatively new technique proposed by Hellerstein and Evans (2017) deemed the ‘Virtual Biopsy’ shows promise in this regard by assessing whole-body muscle mass using metabolic labeling techniques for flux-rate measurements in humans which focus on skeletal muscle, specifically.

When interpreting and contrasting the results of different gross-level measurements, the dimensionality of each needs to be considered. Of course, volumes, areas, and thickness are three-, two-, and one-dimensional, respectively, but understanding what this means in the context of hypertrophy requires deeper thought. Starting from a unidimensional level, muscle can grow in three different, orthogonal directions. For instance, it may be possible that a muscle grows wider but not thicker, or has differential growth in different directions or parts of the muscle. A unidimensional measure, such as thickness via ultrasound, is inherently limited in capturing these possibilities; changes in off-axis lengths will not be captured. Area can, to some extent, account for some of these differences, in that it captures growth across an entire plane. If absolute growth does occur in off-axis directions (e.g., a muscle not only grows thicker, but also wider), or even on parallel axes, we should not expect area to scale linearly with thickness. If thickness predominates, then a linear relationship between thickness and area should hold (Franchi et al., 2018a). Conversely, volume not only takes into account changes in muscle length, but also heterogeneities in area along the muscle. For instance, a muscle may grow more distally than proximally. Such heterogeneities are best captured by taking advantage of the full dimensionality of the construct itself – muscle is three-dimensional. This may be why thickness changes scale with area, but not volume (Franchi et al., 2018a). Unfortunately, our understanding about the dimensionality and heterogeneity of growth itself is limited; these properties may be muscle and protocol-specific.

It is also noteworthy that muscle CSA assessment using imaging techniques can be measured as one of two constructs including ACSA and physiological CSA (PCSA). The former can be assessed at any point along the muscle, and is often defined to be the CSA orthogonal to the longitudinal axis of the segment on which the muscle is located (i.e., in the transverse plane). Alternatively, PCSA is the muscle area orthogonal to a muscle’s fibers; therefore, greater discrepancies will exist between ACSA and PCSA with greater pennation. In contrast to ACSA, PCSA is assessed by measuring muscle volume and dividing it by average fiber length (usually at optimal length). Therefore, PCSA is a measure of the average CSA orthogonal to the fiber orientation and is seemingly related to the number of sarcomeres in parallel (Lieber and Ward, 2011). Although PCSA is more related to a muscle’s function than ACSA, the latter is more commonly reported, primarily due to the methodological limitations of obtaining PCSA.

## Defining Skeletal Muscle Hypertrophy and Future Directions

With intent to provide future research directions and improve the likelihood of fruitful discovery moving forward, it is necessary to operationally define skeletal muscle hypertrophy in an objective manner. To serve as rationale for our proposed definition and types of skeletal muscle hypertrophy, we encourage readers to consider that various types of hypertrophy have been characterized in both cardiac and smooth muscle (Johansson, 1984; Mihl et al., 2008), although the construct of hypertrophy is consistent in these definitions.

We propose that skeletal muscle hypertrophy be generally and simply defined as an increase in skeletal muscle size accompanied by an increase in mineral, protein, or substrate abundance (e.g., glycogen and intramuscular triglyceride). However, considering the reviewed evidence, we encourage the formal adoption of three types of skeletal muscle hypertrophy worthy of further inquiry: (a) connective tissue hypertrophy, (b) myofibrillar hypertrophy, and (c) sarcoplasmic hypertrophy. Connective tissue hypertrophy can be defined as an increase in the volume of the extracellular matrix of skeletal muscle accompanied by an increase in mineral or protein abundance. Sarcoplasmic hypertrophy can be defined as a chronic increase in the volume of the sarcolemma and/or sarcoplasm accompanied by an increase in the volume of mitochondria, sarcoplasmic reticulum, t-tubules, and/or sarcoplasmic enzyme or substrate content. Myofibrillar hypertrophy can be defined as an increase in the size and/or number of myofibrils accompanied by an increase in sarcomere number or sarcomeric protein abundance directly related to the structure or contractile force generation of the sarcomere.

Considering these definitions, we feel it is critical to appreciate the fact that measurement techniques assess different constructs and these differences do not necessarily make a measurement better or worse, but simply different. Resource constraints, technical capabilities, and potential risks to participants are factors that inherently affect the selection of assessments of muscle hypertrophy. To dismiss methodologies that provide less resolution of molecular changes based solely on this fact is inappropriate as good reliability of a test can be incredibly useful for examining changes over time and inferring effects of resistance training interventions. That is to say, while macro- and microscopic tests do not directly assess myofibrillar protein accrual and fCSA, increases in these variables should result in eventual increases in macroscopic indices. Stated differently, while some methods of detecting true hypertrophy are more vivid (e.g., myofibrillar protein), lower resolution methods aren’t useless as they would almost certainly be corollaries of true hypertrophy.

While it can be difficult to reconcile why different methods used to assess skeletal muscle hypertrophy in response to resistance training do not always agree, we posit that certain procedures can be adopted to clarify research findings in the field. First, if a single measure of hypertrophy is being examined (e.g., VL thickness at the mid-thigh) and is found to increase in response to training, then we view reporting that mid-thigh VL thickness increased in response to resistance training rather than stating *muscle hypertrophy* occurred is a more accurate representation of the data. That is, unless multiple levels of measurement are adopted along with assessment of fluid alterations, we encourage the reporting of the measurement itself rather than concluding “skeletal muscle hypertrophy” occurred alone. Alternatively stated, we feel clear language pertaining to the outcome data of a method should be explicitly reported to better portray the nature of a specific measurement. Second, if multiple indices of skeletal muscle hypertrophy are being collected, then it would be valuable to include associations between the measures in order to provide the reader greater insight as to how well or poorly the measurements agree. Third, when possible, we posit that using methods to determine regional fluid shifts that account for ICF and ECF changes could better delineate the mode of hypertrophy and whether mass changes in a region of interest were largely due to fluid accumulation. Certainly, this assumes the research question centers around true protein accretion and not simply an expansion of other microscopic or macroscopic assessments as dependent variables, specifically.

We recently reported ECF-corrected LBM measures to better characterize hypertrophic responses to RT beyond ECF retention potentially due to edema. Although surface electrode BIS possesses limitations given that greater subcutaneous adipose tissue thickness values can negatively influence skeletal muscle impedance readings (Tagliabue et al., 2001), a newer fine needle BIS approach using small subcutaneous needles to bypass subcutaneous fat holds promise for more accurate ICF and ECF assessments (Kwon et al., 2017). Another viable strategy worthy of consideration is to include multiple indices of skeletal muscle hypertrophy at various levels. As an example, our laboratory has implemented K-means cluster analysis based solely upon changes in VL thickness to generate low, moderate, and high anabolic response clusters to 12 weeks of resistance training (Mobley et al., 2018). As discussed earlier, we subsequently adopted a different approach in these same subjects by generating clusters based upon a composite hypertrophy score which entailed percent changes in DXA TBMM (which only considers appendicular lean mass changes), VL thickness using ultrasound, and fCSA, and selected high and low hypertrophic responders whose composite scores existed in the upper and lower quartiles (Roberts M. D. et al., 2018). Notably, in the former publication we did not observe a between-cluster interaction for 3RM squat strength although, as stated above, our latter publication yielded a cluster × time interaction for 3RM squat strength whereby strength gains were greater in high responders. Likewise, a similar multi-variable cluster approach has been adopted by Davidsen et al. (2011), and more recently by Morton et al. (2018) to stratify high-responders and low-responders to resistance training. Thus, if clustering subjects in hopes of characterizing specific phenotypes or predicting adaptive responses thereof is the nature of the research question, we feel using a composite hypertrophy score containing multiple indices (e.g., fCSA + DXA data + limb circumference + muscle thickness, etc.) may be a viable approach. This approach can be thought of as a form of dimensionality reduction; hypertrophic responses can be measured in a number of ways, so procedures such as principal component analysis can yield values that are linear combinations of the predictor variables of interest, and a way that can account for much of the variance. However, as pointed out by Rucker et al. (2015), researchers should thoughtfully consider the specific research question prior to clustering as discretization of continuous data can potentially result in erroneous conclusions. Often, if explaining variation in hypertrophic responses to resistance training is the primary aim via predictors of interest, researchers would likely be better served to include all subjects and their raw, continuous scores in the analysis for greater statistical power.

Finally, calculating the test-retest reliability of hypertrophic assessments to establish standard errors of measurement is also a powerful strategy to more confidently conclude if hypertrophy occurred and direct further exploration. Conceptually, changes beyond calculated measurement error can allow researchers to infer that the specific construct being assessed by the employed technique changed beyond the error of the measurement, regardless of the extent. Often, test-retest reliability is unreported, and depending on the calculated error of measurement, changes within various ranges after resistance training interventions may be better explained by measurement error rather than true variation in the construct being assessed due to the research intervention. As an example, reliability statistics from our laboratory for macroscopic, microscopic, and molecular assessments of hypertrophy are shown in Table 3. Both absolute values (expressed in the unit of the measurement) and relative values (expressed as percentages of the measurement) can be used to construct confidence intervals beyond which changes scores may be more associated with factors other than measurement error. Importantly, even the calculation of measurement error possesses a certain degree of confidence, and this is not posited to negate changes within the calculated range of error, as these changes could still be real. Nevertheless, the combination of the calculated measurement error and the measured change itself provide more information to the researcher, and reader, and can improve the interpretation and presentation of data. Ideally, in the case of a well-controlled resistance training intervention, hypertrophic outcomes could be better associated with the effects of training rather than measurement error itself.

**Table 3:** Test-retest reliability statistics for macroscopic, microscopic, and molecular assessments of hypertrophy.

Variable	*n*	CV of measurement (%)	ICC	Absolute SEM	95% CI	Relative SEM (%)	95% CI (%)
DXA LBM	10	0.95	1.00	0.47 kg	0.92 kg	0.92	1.81
VL thick	30	1.33	0.99	0.04 cm	0.08 cm	1.32	2.59
Mid-thigh circum.	10	0.58	0.99	0.30 cm	0.59 cm	0.57	1.12
Whole-body ICF	30	0.28	1.00	0.08 L	0.16 L	0.27	0.54
Whole-body ECF	30	0.13	1.00	0.02 L	0.05 L	0.13	0.25
Mean fCSA	26	6.65	0.91	459 μm^2^	899 μm^2^	6.59	12.92
MF protein	24	15.60	0.93	38.8 μg/mg^∗^	76.0 μg/mg	15.44	30.27
SARCO protein	24	4.38	0.99	7.5 μg/mg	14.6 μg/mg	4.33	8.49


*These data are from our laboratory (Haun et al., 2018; Roberts M. D. et al., 2018) demonstrating test-retest reliability statistics for macroscopic, microscopic, and molecular assessments of hypertrophy. Abbreviations and symbol: n, n-size included in the analysis (college-aged men only); CV, coefficient of variation; ICC, intra-class correlation coefficient; SEM, standard error of the measurement; DXA LBM, dual x-ray absorptiometry lean body mass; VL thick, ultrasound-assessed vastus lateralis thickness; mid-thigh circum., mid-thigh circumference measured with a tape measure; ICF, intracellular fluid assessed using bioelectrical impedance spectroscopy; ECF, extracellular fluid assessed using BIS; mean fCSA, type I and type II (combined) muscle fiber cross-sectional area; MF protein, myofibrillar protein assessed through biochemical methods; SARCO protein, sarcoplasmic protein assessed through biochemical methods; ^∗^, μg/mg indicates μg pf protein per mg of muscle used for the assay (wet weight).*

To summarize, we propose the following strategies for consideration:

(1) Although we too have underappreciated measurement error previously, we feel a worthwhile future strategy in the hypertrophy literature is to consistently report test-retest reliability statistics and/or calculated standard errors of measurements to better understand changes more associated with training, nutritional, or supplementation interventions. By understanding the expected error of measurement with certain confidence, changes in molecular, microscopic, and macroscopic assessments of muscle hypertrophy can be more accurately surmised with improved confidence.
(2) If molecular levels of measurement are not directly assessed, researchers should report the results of the measurement itself clearly stating the outcome measure in its associated unit instead of invoking the general term hypertrophy alone. For example: “According to DXA, lower extremity lean mass increased by 1 kg.” rather than “According to DXA, muscle hypertrophy occurred.”
(3) Future work can help clarify the specific type of skeletal muscle hypertrophy occurring from an intervention by assessing connective tissue, myofibrillar, and sarcoplasmic fractions of muscle.
(4) Finally, investigators should choose and justify muscle size assessments based on that which best answers their research question. Assessments on different scales are not inherently better or worse, but they are different insofar as their construct validity.

## Conclusion

Assessing the macroscopic, microscopic, and molecular adaptations to resistance training has been a widespread research goal for exercise physiologists since the 19th century. Given the current knowledge-gap regarding the ultrastructural adaptations to resistance training, we hope that future research will better characterize the biochemical and ultrastructural underpinnings of skeletal muscle hypertrophy. While different assessment techniques seem to disagree with one another, we posit that this conundrum provides tremendous opportunity for future researchers to build upon current methods or generate newer and more valid methods to better assess skeletal muscle hypertrophy.

## Data Availability

Publicly available datasets were analyzed in this study. This data can be found at https://peerj.com/articles/5338/#supp-1.

## Author Contributions

CH and MR conceived the discussed topic based on a symposium lecture delivered by MR at the 2018 World Congress of Muscle Hypertrophy at the American College of Sports Medicine Annual Meeting (Minneapolis, MN, United States). CH was the primary architect of this work. All co-authors substantially contributed, edited and approved the final version of this manuscript.

## Conflict of Interest Statement

The authors declare that the research was conducted in the absence of any commercial or financial relationships that could be construed as a potential conflict of interest.

## Footnotes

1 5 sessions per week for 2 weeks consisting of 3 sets of 30 maximal voluntary contractions of the quadriceps muscle.
